# A Novel Conductive Polypyrrole‐Chitosan Hydrogel Containing Human Endometrial Mesenchymal Stem Cell‐Derived Exosomes Facilitated Sustained Release for Cardiac Repair

**DOI:** 10.1002/adhm.202304207

**Published:** 2024-01-14

**Authors:** Changping Yan, Xinzhu Wang, Qi Wang, Haiyan Li, Huifang Song, Jingli Zhou, Zexu Peng, Wenjuan Yin, Xuemei Fan, Kun Yang, Bingrui Zhou, Yuxiang Liang, Zengyu Jiang, Yuwei Shi, Sanyuan Zhang, Sheng He, Ren‐Ke Li, Jun Xie

**Affiliations:** ^1^ The First Hospital of Shanxi Medical University Department of Biochemistry and Molecular Biology Shanxi Key Laboratory of Birth Defect and Cell Regeneration MOE Key Laboratory of Coal Environmental Pathogenicity and Prevention Shanxi Medical University Taiyuan 030001 China; ^2^ Department of Gynecology Affiliated Cancer Hospital of Shanxi Medical University Taiyuan 030013 China; ^3^ Department of Anatomy Shanxi Medical University Taiyuan 030001 China; ^4^ Shanxi Provincial People's Hospital Affiliated Hospital of Shanxi Medical University Taiyuan 030012 China; ^5^ NHC Key Laboratory of Pneumoconiosis Shanxi Province Key Laboratory of Respiratory Department of Pulmonary and Critical Care Medicine The First Hospital of Shanxi Medical University Taiyuan 030001 China; ^6^ Toronto General Hospital Research Institute Division of Cardiovascular Surgery University Health Network University of Toronto Toronto ON M5G 2C4 Canada

**Keywords:** cardiac function, conductive biomaterial, endometrial stem cell, exosome, myocardial infarction

## Abstract

Myocardial infarction (MI) results in cardiomyocyte necrosis and conductive system damage, leading to sudden cardiac death and heart failure. Studies have shown that conductive biomaterials can restore cardiac conduction, but cannot facilitate tissue regeneration. This study aims to add regenerative capabilities to the conductive biomaterial by incorporating human endometrial mesenchymal stem cell (hEMSC)‐derived exosomes (hEMSC‐Exo) into poly‐pyrrole‐chitosan (PPY‐CHI), to yield an injectable hydrogel that can effectively treat MI. In vitro, PPY‐CHI/hEMSC‐Exo, compared to untreated controls, PPY‐CHI, or hEMSC‐Exo alone, alleviates H_2_O_2_‐induced apoptosis and promotes tubule formation, while in vivo, PPY‐CHI/hEMSC‐Exo improves post‐MI cardiac functioning, along with counteracting against ventricular remodeling and fibrosis. All these activities are facilitated via increased epidermal growth factor (EGF)/phosphoinositide 3‐kinase (PI3K)/AKT signaling. Furthermore, the conductive properties of PPY‐CHI/hEMSC‐Exo are able to resynchronize cardiac electrical transmission to alleviate arrythmia. Overall, PPY‐CHI/hEMSC‐Exo synergistically combines the cardiac regenerative capabilities of hEMSC‐Exo with the conductive properties of PPY‐CHI to improve cardiac functioning, via promoting angiogenesis and inhibiting apoptosis, as well as resynchronizing electrical conduction, to ultimately enable more effective MI treatment. Therefore, incorporating exosomes into a conductive hydrogel provides dual benefits in terms of maintaining conductivity, along with facilitating long‐term exosome release and sustained application of their beneficial effects.

## Graphical abstract

Incorporating human endometrial mesenchymal stem cell‐derived exosomes (hEMSC‐Exo) into poly‐pyrrole‐chitosan (PPY‐CHI) yields an injectable hydrogel, possessing conductive and sustained exosome release properties. PPY‐CHI/hEMSC‐Exo counteracts against H_2_O_2_‐induced apoptosis among H9c2, and promotes angiogenesis among human umbilical cord vein endothelial cells. In vivo, it combines the cardiac regenerative capabilities of hEMSC‐Exo with PPY‐CHI conductive properties to alleviate myocardial infarction and arrhythmia.

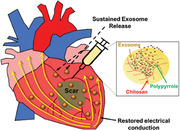

## 1 Introduction

Myocardial infarction (MI) has been identified as a leading cause of cardiovascular disease‐associated death worldwide.^[^ ^1^ ^]^ Current reperfusion strategies reduced mortality at the acute phase^[^ ^2^ ^]^; however, both MI and subsequent reperfusion could lead to vascular endothelial dysfunction, imbalances in the cardiac microenvironment, and myocardial ischemia/reperfusion injury, all of which results in a high risk of heart failure.^[^ ^3^ ^]^ More specifically, the resulting cardiomyocyte necrosis and myocardial fibrosis from MI has been found to be the main contributors behind the development of the 2 major concomitant pathologies of progressive contractive heart failure, as well as cardiac conduction blockages leading to arrhythmia. As a result, tissue regeneration and stem cell therapies to replace the lost cardiomyocytes, as well as pacemakers and cardioversion to restore proper electrical signaling conduction disrupted by fibrosis, have become topics of great interest.

Conductive polymers have emerged as a promising treatment approach for correcting cardiac conduction blockages.^[^ ^4^ ^]^ However, their lower biocompatibility has prevented them from being directly applied to cardiac tissues. Therefore, to overcome this drawback, we have developed a technique to conjugate conductive polymers onto biocompatible molecules, in which poly‐pyrrole (PPY) was first grafted onto chitosan (CHI) to form the bio‐conductive polymer PPY‐CHI,^[^ ^5^ ^]^ which was an injectable, semiconductive, and biocompatible hydrogel able to electrically connect contracting cells at a distance. Furthermore, our in vivo study revealed that PPY‐CHI injection, after MI, decreased the QRS interval and increased the transverse activation velocity, suggesting improved electric conduction.^[^ ^6^ ^]^ The application of PPY‐CHI has been proven to be effective in correcting post‐MI conduction blockage, but they are unable to stimulate tissue regeneration and repair to prevent heart failure.

Accumulated evidence demonstrates that cell therapy fosters cardiac tissue regeneration. One disadvantage, though, is the limited engraftment and survival of transplanted cells, due to the contractile nature of the heart. Therefore, therapies involving stem cell‐derived exosomes have become an attractive alternative approach. Exosomes are small extracellular vesicles, produced by most cell types in the human body, with diameters between 30 and 150 nm.^[^ ^7^ ^]^ In particular, numerous studies have demonstrated that mesenchymal stem cell (MSC)‐derived exosomes are able to improve cardiac functioning post‐MI, by promoting cardiac repair.^[^ ^8^ ^]^ However, rapid clearance of circulating exosomes after systemic injection limits their therapeutic exposure to damaged myocardium.^[^ ^9^ ^]^ Therefore, to improve their therapeutic efficacy and increase retention time at the targeted region, a suitable scaffold system is required to contain these exosomes.^[^ ^10^ ^]^ Injectable hydrogels have been documented to be able to retain exosomes and facilitate their release over a long period of time, and have been demonstrated to be effective in treating MI.^[^ ^11^ ^]^ Furthermore, we have previously demonstrated that human endometrial mesenchymal stem cells (hEMSCs) have significant effects on improving cardiac function, by stimulating angiogenesis and preserving viable cardiomyocytes.^[^ ^12^ ^]^ However, whether it is possible to combine the effects of hEMSCs with the conductive properties of PPY‐CHI to facilitate long‐term exosome release, along with resynchronizing cardiac electrical signaling, has not been fully tested. In this study, we physically crosslinked hEMSC‐derived exosomes (hEMSC‐Exo) with PPY‐CHI hydrogels, and found that as the PPY‐CHI hydrogel degrades, hEMSC‐Exo was able to be slowly released, both in vitro and in vivo, to the MI site. There, the hEMSC‐Exo contents were able to alleviate myocardial tissue damage via activating the epidermal growth factor (EGF)/phosphoinositide 3‐kinase (PI3K)/AKT pathway to promote angiogenesis and inhibit apoptosis. Furthermore, PPY‐CHI/hEMSC‐Exo retained conductive properties, enabling it to alleviate arrhythmia via resynchronizing cardiac electrical transmission.

## 2 Results

### 2.1 Isolation and Characterization of hEMSC‐Exo Properties and Protein Composition

Human uteri tissue was collected from 42 premenopausal women, and hEMSCs isolated using enzymatic digestion. The resulting cultured hEMSCs have typical homogeneous, vortex shapes (Figure S1a, Supporting Information), while alizarin red, Alcian blue, and Oil red O staining confirmed their ability for, respectively, osteogenic, chondrogenic, and adipogenic differentiation (Figure S1b–d, Supporting Information). Furthermore, hEMSCs were found, under flow cytometry, to be positive for CD44, CD73, CD90, and CD105, characteristic of MSCs (Figure S1e, Supporting Information). By contrast, hEMSCs were negative for the hematopoietic surface markers HLA‐DR, CD45, CD19, and CD11b (Figure S1e, Supporting Information).

**Figure 1:**
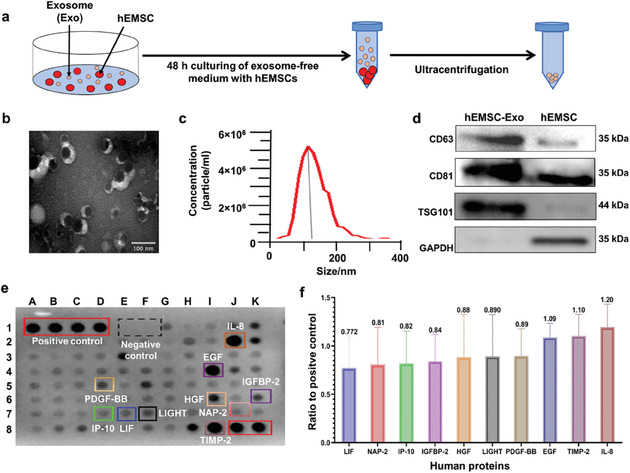
Isolation and characterization of human endometrial mesenchymal stem cell‐derived exosomes (hEMSC‐Exo) properties and protein composition. a) Schematic diagram of hEMSC‐Exo isolation. b) Transmission electron microscope (TEM) micrograph of hEMSC‐Exo. c) Size distribution of hEMSC‐Exo from nanoparticle tracking analysis. d) Western blot image of CD63, CD81, and TSG101 protein expression. The housekeeping gene GAPDH served as a reference. e) Image of human cytokine antibody array. f) Top 10 proteins expressed by hEMSC‐Exo, as determined by the human cytokine antibody array. Data are expressed as mean ± standard deviation (SD). *n* = 9/group for c), *n* = 3/group for f).

hEMSCs were cultured in Dulbecco's modified Eagle's medium (DMEM)/Ham's Nutrient Mixture F‐12, and hEMSC‐Exo was isolated from the resulting conditioned media with ultracentrifugation (**Figure** **1**a). The isolated exosomes displayed the typical discoid vesicular structure under transmission electron microscopy (TEM; Figure 1b). The average size of hEMSC‐Exo was found under nanoparticle tracking analysis (NTA) to be 130 ± 4.37 nm (Figure 1c), and they were positive for specific exosome markers CD63, CD81, and TSG101 under Western blot (Figure 1d). To further identify hEMSC‐Exo contents, the human cytokine antibody array was used to identify highly‐expressed proteins, as shown in Figure 1e. The top 10 most‐expressed proteins from these exosomes were, from lowest to highest, leukemia inhibitory factor (LIF), neutrophil activating peptide 2 (NAP‐2), interferon‐γ‐induced protein 10 (IP‐10), insulin‐like growth factor binding protein 2 (IGFBP‐2), hepatocyte growth factor (HGF), tumor necrosis factor superfamily member 14 (TNFSF14/LIGHT), platelet‐derived growth factor BB (PDGF‐BB), EGF, tissue inhibitor of metalloproteinases 2 (TIMP‐2), and interleukin 8 (IL‐8; Figure 1f). These findings therefore confirmed the successful isolation of hEMSC‐Exo, which was enriched with a wide variety of biological molecules.

### 2.2 hEMSC‐Exo Inhibited H_2_O_2_‐Induced H9c2 Cell Apoptosis and Facilitated Endothelial Cell Angiogenesis

**Figure 2:**
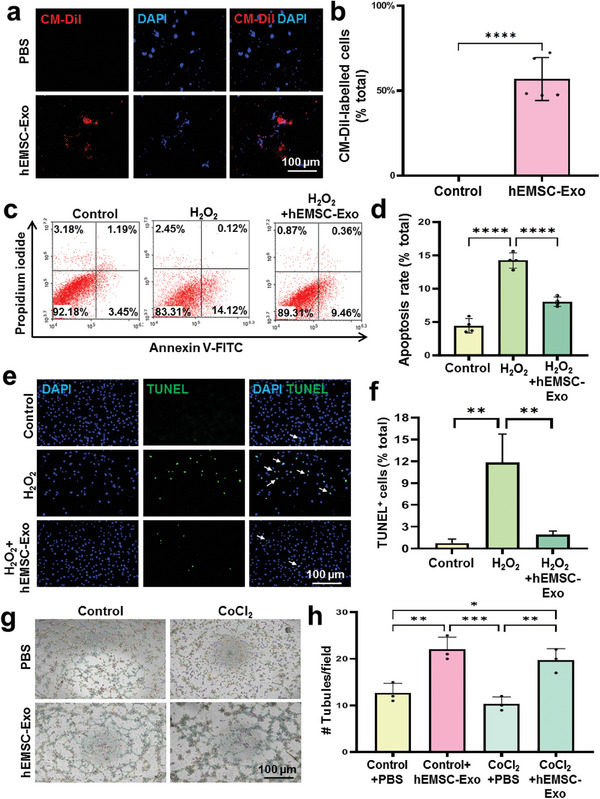
hEMSC‐Exo inhibited H_2_O_2_‐induced apoptosis of H9c2 cells and facilitated human umbilical cord vein endothelial cell (HUVEC) angiogenesis. a) Representative images and b) quantification showing successful uptake of CM‐Dil‐labeled (red) hEMSC‐Exo among H9c2 cells, compared to PBS‐treated control H9c2 cells. Nuclei were stained with 4′, 6‐diamidino‐2‐phenylindole (DAPI; blue). c) Flow cytometry and d) quantification for Annexin V^+^ H9c2 cells, indicating the occurrence of apoptosis, among untreated control (Control), H_2_O_2_‐treated (H_2_O_2_, 900 µm), and H_2_O_2_+hEMSC‐Exo (10 µg mL^−1^) groups. e) Representative TUNEL staining images for apoptotic cells (green) and f) quantification of apoptotic cell rates among the 3 groups. g) Representative micrographs and h) quantification of Matrigel tube formation among PBS‐treated HUVECs that are either nonhypoxic (Control+PBS) or cotreated with CoCl_2_ (hypoxia inducer, 400 µm; CoCl_2_+PBS), as well as nonhypoxic (Control+hEMSC‐Exo) or CoCl_2_‐treated HUVECs cotreated with hEMSC‐Exo (CoCl_2_+hEMSC‐Exo, 200 µg mL^−1^) groups. Data are expressed as mean ± SD. *n* = 5–6/group for b,d), *n* = 3/group for f,h). *p < 0.05, **p < 0.01, ***p < 0.001, ****p < 0.0001.

It has been documented that the uptake of exosomes is a critical step for exerting their effects.^[^ ^11b^ ^]^ To confirm hEMSC‐Exo uptake by cells, CM‐Dil‐labeled hEMSC‐Exo were cocultured with H9c2 cells for 6 h. After 6 h, red fluorescence was observed within the cytoplasm of H9c2 cells treated with hEMSC‐Exo, while control cells treated with PBS did not have any fluorescence present, indicating that hEMSC‐Exo was take up by H9c2 cells (**Figure** **2**a,b).

To investigate if hEMSC‐Exo protects H9c2 myoblasts from reactive oxygen species (ROS) exposure, myoblasts were treated with 900 µm H_2_O_2_ for 4 h, followed by 10 µg mL^−1^ of hEMSC‐Exo for 24 h. We found, based on flow cytometry quantification of Annexin V^+^ cells and TUNEL staining, that hEMSC‐Exo‐treated myoblasts had significantly lower percentages of apoptotic cells, compared to H_2_O_2_‐treated ones, indicating that the exosomes exerted cyto‐protective effects on those myoblasts (Figure 2c–f).

We also examined whether hEMSC‐Exo can stimulate pro‐angiogenic activities with human umbilical cord vein endothelial cells (HUVECs). HUVECs were first exposed to CoCl_2_, a hypoxia inducer,^[^ ^13^ ^]^ for 24 h, followed by treatment with 200 µg mL^−1^ hEMSC‐Exo for 3.5 h. Matrigel tubule assay demonstrated that hEMSC‐Exo‐treated HUVECs, compared to untreated control cells, had significantly greater number of tubular structures, under both hypoxic and nonhypoxic conditions (Figure 2g,h). Therefore, hEMSC‐Exo treatment was able to counteract ROS‐induced myoblast apoptosis, as well as promoting angiogenesis among HUVECs.

### 2.3 hEMSC‐Exo Exerts Cyto‐Protective Effects via the EGF/PI3K/AKT Pathway

Exosome contents serve as significant mediators for various biological functions. Therefore, to identify which of the 10 most‐expressed proteins in hEMSC‐Exo was responsible for the antiapoptotic and proangiogenic effects of these exosomes, transcriptome sequencing was performed, in which 10 of the most‐expressed miRNAs were associated with EGF/EGFR proteins (Figure S2a, Supporting Information). These genes were associated with angiogenic and antiapoptotic functions (Figure S2b,c, Supporting Information). Kyoto Encyclopedia of Genes and Genomes (KEGG) have found those genes to be most enriched for functions related to PI3K/AKT signaling (Figure S2d, Supporting Information), which was further verified by the EGF protein network diagram (Figure S2e, Supporting Information).

**Figure 3:**
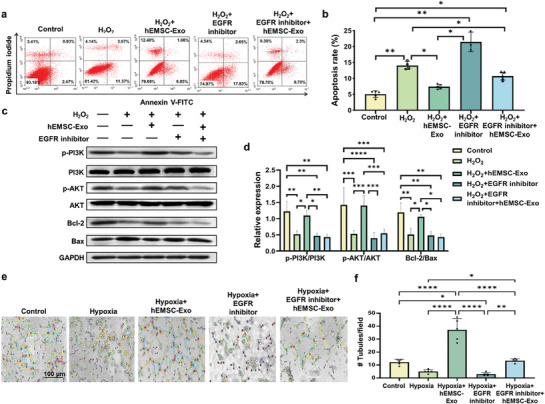
hEMSC‐Exo exerts cyto‐protective effects via the EGF/PI3K/AKT pathway. a) Flow cytometry and b) quantification for Annexin V^+^ H9c2 cells, indicating the occurrence of apoptosis, among untreated control (Control), H_2_O_2_‐treated (H_2_O_2_), H_2_O_2_+hEMSC‐Exo, H_2_O_2_+EGFR inhibitor, and H_2_O_2_+EGFR inhibitor+hEMSC‐Exo groups. c) Western blot images and d) quantification of phospho‐(p)‐PI3K, PI3K, p‐AKT, AKT, Bcl‐2 (anti‐apoptotic), and Bax (pro‐apoptotic) protein expression. The housekeeping gene GAPDH served as a reference. e) Representative micrographs and f) quantification of Matrigel tube formation by HUVECs among untreated control (Control), hypoxic (Hypoxia), Hypoxia+hEMSC‐Exo, Hypoxia+EGFR inhibitor, and Hypoxia+EGFR inhibitor+hEMSC‐Exo groups. Data are expressed as mean ± SD. *n* = 3–5/group for b), *n* = 3/group for d,f). *p < 0.05, **p < 0.01, ***p < 0.001, ****p < 0.0001.

EGF has been documented to be associated with antiapoptotic activities, via binding to the receptor tyrosine kinase EGFR, and subsequently activating the PI3K/AKT pathway.^[^ ^14^ ^]^ To further confirm whether hEMSC‐Exo exerted its cyto‐protective effects via the EGF/PI3K/AKT pathway, 5 groups of myoblasts were examined under flow cytometry for Annexin V^+^ cells: untreated control (Control), H_2_O_2_‐treated (H_2_O_2_), H_2_O_2_+hEMSC‐Exo, H_2_O_2_+EGFR inhibitor, and H_2_O_2_+EGFR inhibitor+hEMSC‐Exo (**Figure** **3**a). We found that compared to Control, H_2_O_2_ treatment resulted in a significantly higher percentage of apoptotic cells. However, H_2_O_2_+hEMSC‐Exo reversed apoptosis levels back toward that of Control. On the other hand, administration of EGFR inhibitors yielded apoptosis rates even greater than that of H_2_O_2_. These levels, were lowered, though not to the levels of Control, in the H_2_O_2_+EGFR inhibitor+hEMSC‐Exo group (Figure 3b), suggesting that EGFs may serve as mediators in antiapoptotic processes. Similar results were also observed under hypoxic conditions for the following 5 groups of H9c2 cells: Control, Hypoxia, Hypoxia+hEMSC‐Exo, Hypoxia+EGFR inhibitor, and Hypoxia+EGFR inhibitor+hEMSC‐Exo. There, compared to Control, Hypoxia had significantly increased apoptotic cell counts, as determined by TUNEL staining, However, Hypoxia+hEMSC‐Exo, reversed apoptosis levels back toward that of Control. On the other hand, the addition of EGFR inhibitor yielded apoptosis rates greater to that of Hypoxia, which was unable to be completely reversed in the presence of hEMSC‐Exo, indicating that hEMSC‐Exo was able to exert cytoprotective effects under hypoxia, probably via the same mediators as for H_2_O_2_ treatment (Figure S3, Supporting Information).

To verify whether hEMSC‐Exo/EGF operated through the PI3K/AKT pathway, protein expression levels were examined from the 5 groups of myoblasts. We found that for phosphorylated (p)‐AKT and p‐PI3K, H_2_O_2_ treatment significantly decreased expression levels compared to Control, However, H_2_O_2_+hEMSC‐Exo reversed these levels back toward that of Control. Conversely, EGFR inhibitors yielded similar expression levels to that of H_2_O_2_, no matter the presence of hEMSC‐Exo (Figure 3c,d). Western blot was also used to further examine the downstream mediators of apoptosis, antiapoptotic Bcl‐2 and pro‐apoptotic Bax, in which the same trends for p‐AKT and p‐PI3K were observed for the Bcl‐2/Bax ratio; the highest levels were found for Control and H_2_O_2_+hEMSC‐Exo groups (Figure 3c,d). These findings thus demonstrate that the anti‐apoptotic effects of hEMSC‐Exo were most likely exerted via the EGF/PI3K/AKT pathway.

To investigate whether EGF was involved in tubule formation among HUVECs, 5 cell groups were examined: untreated control (Control), Hypoxia, Hypoxia+hEMSC‐Exo, Hypoxia+EGFR inhibitor, and Hypoxia+EGFR inhibitor+hEMSC‐Exo (Figure 3e). We found that compared to Control, Hypoxia and Hypoxia+EGFR inhibitor had lower numbers of tubules formed. However, Hypoxia+hEMSC‐Exo tubule formation was much greater than all the other groups. It is worth noting, though, that co‐treatment of EGFR inhibitor with hEMSC‐Exo among hypoxic HUVECs still reversed tubule formation back toward that of Control (Figure 3f). These observations therefore suggest that hEMSC‐Exo also exerted pro‐angiogenic effects on hypoxic HUVECs.

### 2.4 Incorporation of hEMSC‐Exo into PPY‐CHI Hydrogel Ensures Sustained Exosome Release, While Maintaining Electrical Conductivity

**Figure 4:**
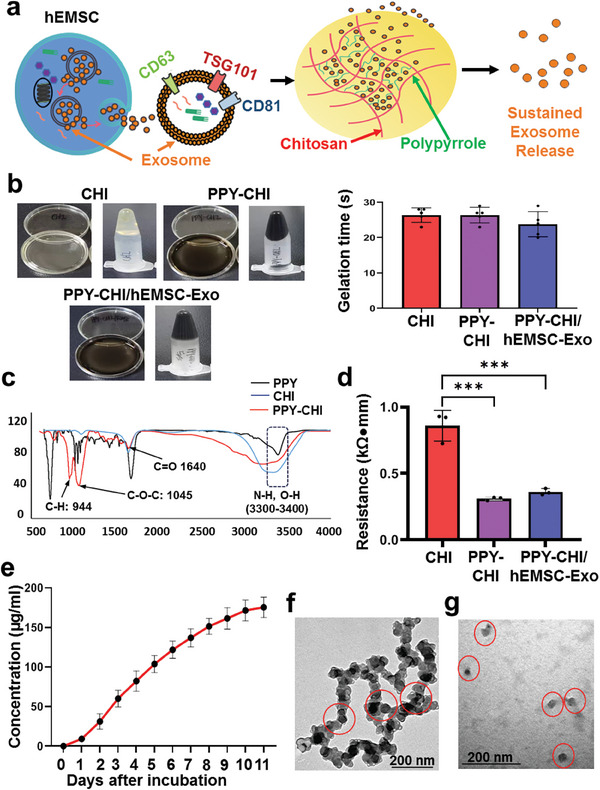
hEMSC‐Exo incorporation into polypyrrole‐chitosan (PPY‐CHI) hydrogel ensures sustained exosome release, while maintaining electrical conductivity. a) Schematic illustration of hEMSC‐Exo incorporation into the PPY‐CHI hydrogel. b) Images of CHI, PPY‐CHI, and PPY‐CHI/hEMSC‐Exo postgelation, in which they adhered to the bottom of the dish and Eppendorf tube, as well as quantification of gelation times. c) Fourier‐transform infrared (FT‐IR) spectra for PPY, PPY‐CHI, and CHI, showing successful conjugation PPY on CHI. d) Resistance among CHI, PPY‐CHI, and PPY‐CHI/hEMSC‐Exo. e) Cumulative release of proteins from PPY‐CHI/hEMSC‐Exo over the course of 11 days. Representative TEM micrographs of f) hEMSC‐Exo in PPY‐CHI hydrogel at day 4 and g) hEMSC‐Exo within the supernatant at day 7. Data are expressed as mean ± SD. *n* = 5/group for b), *n* = 3/group for c,d,e). ***p < 0.001.

In order to yield the maximal beneficial effects for hEMSC‐Exo for cardiac applications post‐MI, we have incorporated them into the PPY‐CHI hydrogel for sustained release, as shown in the schematic in **Figure** **4**a. The resulting PPY‐CHI/hEMSC‐Exo hydrogel has a number of advantages, one of which is enabling slow, sustained release of exosomes, and another is the capability to conduct cardiac electrical signals, yielding a synergistic treatment approach facilitating biological regeneration and conductive system repair. We have previously characterized PPY‐CHI, with respect to different polymer versus CHI ratios, as well as its biocompatibility, and found that 3:10 PPY:CHI was the optimal ratio for forming a semiconductive hydrogel, with no toxic effects on in vitro smooth muscle cell and cardiomyocyte growth, proliferation, or metabolic activities.^[^ ^5^, ^6^ ^]^ With respect to hEMSC‐Exo incorporation, we also found that it did not significantly affect the gelation properties of the PPY‐CHI/hEMSC‐Exo, which were similar to that of CHI and PPY‐CHI (Figure 4b). Furthermore, no significant differences, in terms of cell viability, was present for H9c2 cells and HUVECs grown on CHI, PPY‐CHI, and PPY‐CHI/hEMSC‐Exo‐coated plates, compared to that of control (Figure S4, Supporting Information). All these observations thus reinforced the finding that PPY‐CHI, as well as PPY‐CHI/hEMSC‐Exo, was biocompatible, with low cytotoxicity.

Successful conjugation of PPY with CHI was verified using Fourier‐transform infrared spectroscopy (FT‐IR), as shown in Figure 4c, in which for PPY, the peaks observed at 3394 and 1014 cm^−1^ represent N*─*H and C*─*H stretching vibrations, while the C*─*N stretching vibration is represented by a peak at 1415 cm^−1^, and the C═C stretching vibration by a peak at 1670 cm^−1^. For CHI, the peaks at 975 and 1086 cm^−1^ represent the C*─*O stretching vibration, while 1636 cm^−1^ represents C*─*OH bending vibration. PPY to CHI conjugation was confirmed by the presence of a peak corresponding to C═O bonds, and thus the acetylated amino group, at 1640 cm^−1^. Peaks were also observed at 944 and 1045 cm^−1^, representing, respectively, aromatic C*─*H out‐of‐plane bending and C*─*O*─*C tensile vibrations. Furthermore, the composite spectra showed wide absorption peaks at 3300–3400 cm^−1^, representing N*─*H stretching vibrations of PPY, and O*─*H stretching vibrations of CHI. All these analyses showed that PPY was indeed conjugated onto the CHI backbone.

To verify whether hEMSC‐Exo incorporation affected the electrical conductivity of the PPY‐CHI hydrogel, the resistance was measured, in which no significant difference was present between PPY‐CHI and PPY‐CHI/hEMSC‐Exo; both had significantly lower resistances compared to CHI (Figure 4d), suggesting that addition of hEMSC‐Exo did not alter the conductive properties of PPY‐CHI. We also examined the release of proteins from PPY‐CHI/hEMSC‐Exo over time, in which a step‐wise increase in released proteins was present over the course of 11 days (Figure 4e). Furthermore, to determine whether hEMSC‐Exo were able to be released intact from PPY‐CHI/hEMSC‐Exo, supernatant was collected daily for 11 days, which was then centrifuged and examined under TEM. We found that the exosomes released from PPY‐CHI/hEMSC‐Exo still displayed the typical discoid vesicular structure over the 11‐day period (Figure 4f,g). Therefore, the usage of glutaraldehyde in the conjugation of PPY with CHI did not affect exosome bio‐stability, as they were still able to be released from the hydrogel, with intact vesicular structures. Ultimately, PPY‐CHI/hEMSC‐Exo was able to foster slow, sustained release of exosome proteins, while maintaining its conductivity.

### 2.5 PPY‐CHI/hEMSC‐Exo Implantation Improved Post‐MI Cardiac Functioning, Along with Reducing Ventricular Remodeling and Fibrosis

**Figure 5:**
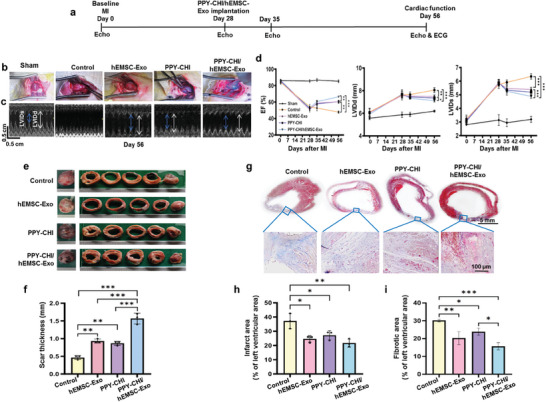
PPY‐CHI/hEMSC‐Exo injection improved postmyocardial infarction (MI) cardiac functioning, along with reducing ventricular remodeling and fibrosis. a) Schematic showing the timeline for MI induction, PPY‐CHI/hEMSC‐Exo injection, and cardiac functional evaluation, over the course of 56 days. b) Images demonstrating successful establishment of phosphate buffer saline (PBS Control), hEMSC‐Exo, PPY‐CHI and PPY‐CHI/hEMSC‐Exo rat groups. c) Representative M‐mode echocardiography images taken on day 56 post‐MI among the 5 groups. d) Measurements for ejection fraction (EF), as well as left ventricular internal diameter end diastole (LVIDd) and systole (LVIDs) among the 5 groups. e) Representative images and f) ventricular wall thickness measurements of rat heart tissue sections, obtained at 4 weeks postinjection, among the 4 groups. g) Representative Masson trichrome staining images for the 4 treatment groups. Quantification of h) infarcted and i) fibrotic areas among the 4 treatment groups. Data are expressed as mean ± SD. *n* = 6/group for d), *n* = 3/group for f,h,i), *p < 0.05, **p < 0.01, ***p < 0.001.

We then evaluated the effect of PPY‐CHI/hEMSC‐Exo in vivo via implantation into infarct myocardium of adult rat heart post coronary arterial ligation. **Figure** **5**a shows the timeline of MI induction, PPY‐CHI/hEMSC‐Exo injection, and cardiac functional evaluation, over the course of 56 days. Five experimental groups were established: Sham, Control (PBS), as well as hEMSC‐Exo, PPY‐CHI or PPY‐CHI/hEMSC‐Exo injection into the infarct site and surrounding border areas at 28 days post‐MI (Figure 5b). Echocardiography was then carried out, and representative M‐mode images were taken on day 56 (Figure 5c). We found no difference at baseline prior to MI among the 5 groups, with respect to ejection fraction (EF), as well as left ventricular internal diameter end diastole (LVIDd), and systole (LVIDs). Additionally, on day 28 post‐MI, EF significantly decreased, while LVIDd and LVIDs increased among all groups except Sham, indicating the occurrence of post‐MI‐induced ventricular dilation. However, from day 35, or 7 days after biomaterial implantation, until the end of the study period on day 56, the highest EF was present among PPY‐CHI/hEMSC‐Exo, Control the lowest, and PPY‐CHI and hEMSC‐Exo in between. Conversely, for LVIDd and LVIDs, Control had the highest value, PPY‐CHI/hEMSC‐Exo the lowest, and PPY‐CHI and hEMSC‐Exo in between (Figure 5d).

The rat hearts were then studied histologically 4 weeks post‐biomaterial injection. We found that the 3 treatment groups had significantly thicker ventricular walls, compared to PBS control, with PPY‐CHI/hEMSC‐Exo having the thickest walls, under morphometric analysis (Figure 5e,f). The size of the infarcted area and fibrotic regions of the hearts for the 4 groups was then measured using Masson trichrome staining, in which the 3 treatment groups had smaller infarcted and fibrotic areas, compared to that of Control; the smallest areas were found among PPY‐CHI/hEMSC‐Exo (Figure 5g–i), indicating that exosome administration, particularly when incorporated into PPY‐CHI, was able to alleviate post‐MI ventricular remodeling and reduce fibrosis.

### 2.6 PPY‐CHI/hEMSC‐Exo Injection Alleviated Arrhythmia

**Figure 6:**
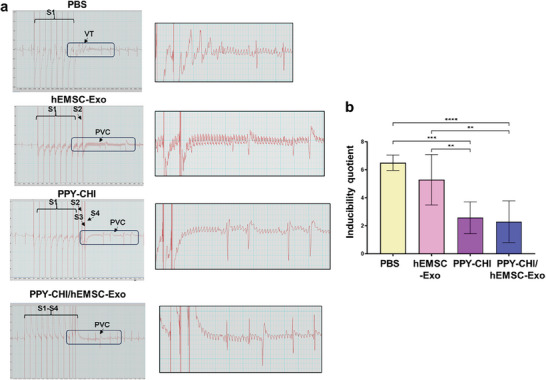
PPY‐CHI/hEMSC‐Exo injection alleviated arrhythmia. a) Representative programmed electrical stimulation‐induced arrhythmias among PBS Control, hEMSC‐Exo, PPY‐CHI, or PPY‐CHI/hEMSC‐Exo rat groups, at 4 weeks postbiomaterial injection (Day 56). S1 represented 8‐burst stimulation, S2 16‐burst stimulation, S3 32‐burst stimulation, and S4 64‐burst stimulation. VT represented ventricular tachycardia and PVC premature ventricular contractions. b) Induction quotient scores, representing arrhythmia susceptibility, among the 4 groups; higher scores corresponded to greater susceptibility. Data are expressed as mean ± SD. *n* = 3–9/group for b), **p < 0.01, ***p < 0.001, ****p < 0.0001.

To determine whether PPY‐CHI/hEMSC‐Exo could alleviate arrhythmia in the MI hearts, programmed electrical stimulation (PES)^[^ ^15^ ^]^ was used to induce arrhythmias in MI rats 4 weeks postbiomaterial injection. Electrocardiography (ECG) traces from the 4 groups demonstrated successful induction in the form of premature ventricular contractions (PVCs; **Figure** **6**a). Based on induction quotients, the highest scores were present among PBS control and hEMSC‐Exo, indicating greater susceptibility to arrhythmia. By contrast, PPY‐CHI and PPY‐CHI/hEMSC‐Exo had lower scores, and thus lower susceptibility (Figure 6b). All these findings therefore indicated that PPY‐CHI/hEMSC‐Exo was able to improve post‐MI cardiac functional parameters and alleviate arrhythmia in vivo.

### 2.7 PPY‐CHI/hEMSC‐Exo Exerted Proangiogenic and Antiapoptotic Effects In Vivo via the EGF/PI3K/AKT Pathway

**Figure 7:**
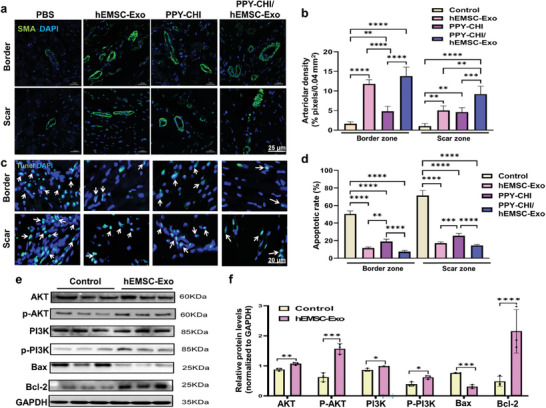
PPY‐CHI/hEMSC‐Exo exerted proangiogenic and antiapoptotic effects in vivo via the EGF/PI3K/AKT pathway. a) Representative immunofluorescence images for arteriolar density, in the form of α‐smooth muscle actin (SMA)^+^ areas (green), and b) quantification of arteriolar densities, for both border and scar zones of the infarcted region, among PBS Control, hEMSC‐Exo, PPY‐CHI, and PPY‐CHI/hEMSC‐Exo groups. Nuclei were stained with DAPI (blue). c) Representative TUNEL staining images for apoptotic cells (green), and d) quantification of apoptotic cell rates among the 4 groups, in both border and scar zones. e) Western blot image and f) quantification of AKT, p‐AKT, PI3K, p‐PI3K, Bax, and Bcl‐2 protein expression levels. The housekeeping gene GAPDH served as a reference. Data are expressed as mean ± SD. *n* = 5/group for b,d), *n* = 3/group for f). *p < 0.05, **p < 0.01, ***p < 0.001, ****p < 0.0001.

To further verify whether the in vitro effects of hEMSC‐Exo in promoting angiogenesis and counteracting against apoptosis was also present in vivo for PPY‐CHI/hEMSC‐Exo, immunofluorescence staining was used to measure arteriolar densities, in the form of α‐smooth muscle actin (α‐SMA)^+^ areas (**Figure** **7**a). We found that among both border and scar regions, the 3 treatment groups had significantly higher arteriolar densities compared to PBS control, with PPY‐CHI/hEMSC‐Exo having the highest density (Figure 7b). Apoptotic cell counts within the infarcted scar and border regions of cardiac tissues was then measured using TUNEL staining (Figure 7c), where it was found that these 3 groups had significantly lower apoptotic cell counts versus Control, with the exosome‐treated groups having lower counts compared to PPY‐CHI (Figure 7d). This was consistent with our in vitro evaluations, in which 3 groups of H9c2 cells were examined: Hypoxia+hEMSC‐Exo, Hypoxia+PPY‐CHI, and Hypoxia+PPY‐CHI/hEMSC‐Exo. We found that hypoxia treatment resulted in significantly higher apoptotic cell numbers on PPY‐CHI‐coated dishes, while Hypoxia+hEMSC‐Exo and Hypoxia+PPY‐CHI/hEMSC‐Exo had protective effects against hypoxia‐induced apoptosis, as indicated by TUNEL staining (Figure S5a,b, Supporting Information). Additionally, HUVEC tube formation was evaluated in the following 3 groups, using CoCl_2_: CoCl_2_+hEMSC‐Exo, CoCl_2_+PPY‐CHI, and CoCl_2_+PPY‐CHI/hEMSC‐Exo, in which the number of tubes formed in both CoCl_2_+hEMSC‐Exo and CoCl_2_+PPY‐CHI/hEMSC‐Exo was comparable to each other, and much greater than that of the CoCl_2_+PPY‐CHI (Figure S5c,d, Supporting Information). All these findings suggested that the prosurvival and proangiogenic effects were most likely due to the presence of hEMSC‐Exo within the hydrogel, rather than PPY‐CHI itself. Therefore, the same proangiogenic and antiapoptotic effects hEMSC‐Exo exerted in vitro was also present in vivo when incorporated as part of the PPY‐CHI hydrogel. These effects were also found to involve the EGF/PI3K/AKT pathway, as cardiac tissues treated with exosomes, compared to untreated controls, had significantly higher protein expression levels for AKT, p‐AKT, PI3K, p‐PI3K, and antiapoptotic Bcl‐2, as well as lower levels for proapoptotic Bax (Figure 7e,f). All these findings thus demonstrate that PPY‐CHI/hEMSC‐Exo, in vivo, was able to yield the same proangiogenic and antiapoptotic effects as hEMSC‐Exo in vitro, most likely exerted via the same EGF/PI3K/AKT pathway.

## 3 Discussion

MI is usually caused by coronary artery occlusion, in turn contributing to subsequent myocardial ischemia and tissue necrosis.^[^ ^16^ ^]^ All these pathological processes contribute to the high mortality; thus, counteracting against ischemia‐associated cardiomyocyte apoptosis and remediating oxygen supply‐demand imbalances via promoting myocardial vascularization have been considered as the main approaches for treating MI.^[^ ^17^ ^]^ hEMSCs have been reported in our previous study to be able to contribute to such antiapoptotic and proangiogenic processes, and subsequently improve post‐MI cardiac functioning, with greater effectiveness than that of bone marrow MSCs.^[^ ^12a^ ^]^ However, cell therapies, due to the potential for immunological rejection, have had significant limitations for widespread clinical applications. Thus, MSC‐derived exosomes have become an increasingly popular approach, as they could potentially yield similar outcomes to that of stem cell therapies, but devoid of immunogenic barriers. One disadvantage of exosome therapy, though, is that exosomes have a short half‐life within the human body, limiting the application of their beneficial effects over longer timespans.^[^ ^18^ ^]^ Furthermore, the effects of hEMSC‐derived exosomes for treating MI have not been fully examined,^[^ ^19^ ^]^ and post‐MI electrophysiological abnormalities, due to fibrosis, cannot be corrected by those exosomes. Therefore, in this study, we aimed to remediate these shortcomings by examining the effectiveness of the novel PPY‐CHI/hEMSC‐Exo hydrogel, comprising hEMSC‐Exo conjugated to the conductive hydrogel PPY‐CHI,^[^ ^15a^ ^]^ on treating MI.

Both in vitro and in vivo, PPY‐CHI/hEMSC‐Exo was found to be able to lower apoptosis and promote proangiogenic activities among, respectively, ROS‐exposed H9c2 and hypoxic HUVEC cells, as well as in a rat MI model, all of which were in line with observations from previous studies.^[^ ^20^ ^]^ More specifically, hEMSC exosome treatment with H9c2 and HUVECs resulted in lowered apoptotic cell counts among the former, as well as increased Matrigel tubule formation among the latter. As for the in vivo model, PPY‐CHI/hEMSC‐Exo was able to alleviate arrhythmia via resynchronizing myocardial electrical conduction, along with counteracting against post‐MI ventricular remodeling via increasing ventricular wall thickness and reducing the fibrotic area present in the infarcted region. These activities, in turn, contributed to improved cardiac functional parameters at the end of the 4‐week treatment period.

For biomaterial applications in cell therapies, research has focused on ones able to provide a temporary matrix for cell attachment, such as collagen,^[^ ^21^ ^]^ alginate,^[^ ^22^ ^]^ fibrin,^[^ ^23^ ^]^ and matrigel.^[^ ^24^ ^]^ All of these studies demonstrated improved cell survival, along with preservation of left ventricular geometry and function. However, conduction through the infarcted region has remained a problem, as the injected cells are poorly distributed as clusters within the scar tissue, possibly yielding a proarrhythmic heterogeneous milieu.^[^ ^25^ ^]^ Therefore, there is an unmet demand for a biomaterial, which not only provides a matrix for cell attachment, but is also able to correct conduction abnormalities. Conductive polymers were first described in 1977, and are particularly appealing due to their electric properties as semiconductors, along with retaining ease of processing, and modifiable conductivity.^[^ ^26^ ^]^ However, poor biocompatibility associated with these polymers limits their usage for cardiac repair. As a result, we developed a technique to conjugate conductive polymers onto biocompatible molecules to form conductive‐biomaterials, such as PPY‐CHI.^[^ ^5^ ^]^ Increasing PPY quantities in PPY‐CHI resulted in increased electrical conductivities, and cardiomyocyte contraction synchronization. These PPY polymers were also found to be evenly distributed within CHI, yielding uniform conductivity throughout scaffold of the biomaterial. Using a rat myocardial infarction (MI) model, we demonstrated that PPY‐CHI injection into the infarct decreased QRS intervals, along with increasing transverse activation velocities and action potential amplitudes, compared to CHI control.^[^ ^5^, ^15^ ^]^ Additionally, PPY‐CHI was still visible at the infarct zone for at least 2–3 months postinjection,^[^ ^5^, ^15^ ^]^ indicating that PPY was able to be retained within the cardiac tissue to continue to influence electrical conduction. Based on those previous findings, we took a further step by combining the synergistic effects of PPY‐CHI and hEMSC‐Exo to form a novel hydrogel, which facilitated sustained release of tissue regenerative hEMSC‐Exo, along with correcting conductive abnormalities.

To examine the underlying mechanistic bases behind PPY‐CHI/hEMSC‐Exo being able to counteract against apoptosis and promote angiogenesis in treating MI, we examined the exosome contents to identify key proteins and RNA sequences, using, respectively, the human cytokine antibody array and transcriptome sequencing. We found that the most likely protein involved in these aforementioned MI‐alleviating processes was EGF, while KEGG analysis found these transcripts to be most enriched for the PI3K/AKT pathway. Both of these findings were in line with the literature,^[^ ^27^ ^]^ which associated PI3K/AKT pathway activation, triggered by EGF, with proangiogenic and antiapoptotic processes.^[^ ^28^ ^]^ Indeed, both in vitro and in vivo, PPY‐CHI/hEMSC‐Exo was associated with increased protein expression of activated p‐PI3K and p‐AKT proteins. These findings were further confirmed with cotreatment of hEMSC‐Exo‐treated ROS‐exposed H9c2 cells and hypoxic HUVECs with EGFR inhibitors, which reversed the hEMSC‐Exo‐associated increases in p‐PI3K and p‐AKT, and subsequently the antiapoptotic and angiogenesis‐favoring effects. All these findings therefore demonstrate that EGF/PI3K/AKT is most likely the key pathway responsible for the effects of PPY‐CHI/hEMSC‐Exo.

MSC‐derived exosomes have been documented to play important roles in cardiovascular disease and development, via their involvement in proangiogenic, anti‐inflammatory, and antiapoptotic, as well as other immune responses.^[^ ^29^ ^]^ For instance, one of the miRNAs found within these exosomes is miR‐486‐5p, which has been documented to promote angiogenesis and subsequent cardiac functional recovery, without aggravating existing arrhythmias, in a nonhuman primate MI model.^[^ ^30^ ^]^ On top of promoting angiogenesis and counteracting against apoptosis and inflammation, MSCs and their products have also been observed to possess reparative capabilities post‐MI, as shown in a study in which hydrogels containing pluripotent and mesenchymal stem cells, after being injected into the pericardial cavity, demonstrated such capabilities in a rat MI model.^[^ ^31^ ^]^ These capabilities were also exerted by hEMSC exosome contents, such as KLF‐AS1, which was able to reduce the MI infarct area, particularly via reducing apoptosis and pyroptosis; this is owed to it being able to act as a “sponge” against miR‐138‐5p and by activating Sirt1.^[^ ^32^ ^]^ All of these observations were thus in line with our findings demonstrating that EGF from hEMSC‐Exo was able to promote MI‐alleviating activities via activating PI3K/AKT signaling. These findings further highlighted that all of these proangiogenic and proreparative, as well as anti‐inflammatory and antiapoptotic properties, from MSC‐derived exosomes could be exerted over longer time periods if these exosomes were incorporated within hydrogels; the resulting sustained release, in turn, facilitated long‐term alleviation of MI and cardiac functional improvements. However, one significant limitation is that our transcriptome sequencing only revealed one involved pathway, the EGF/PI3K/AKT pathway, based on the most highly‐expressed miRNAs; other possible genes and pathways involved have not been fully investigated, which will be the subject of future studies, in order to fully optimize the hydrogels to aid in MI treatment.

## 4 Conclusion

In summary, the data from this study support that PPY‐CHI/hEMSC‐Exo, a conductive hydrogel with long‐term sustained release of exosome contents, could aid in alleviating MI and improving cardiac functioning. In vitro, hEMSC‐Exo was found to counteract against apoptosis among H9c2 cells, and promote angiogenesis‐favoring activities among HUVECs, while in vivo, these antiapoptotic and proangiogenic processes, along with alleviating arrhythmia via resynchronizing cardiac electrical conduction, was conducted by PPY‐CHI/hEMSC‐Exo in the rat MI model. All of these functions were most likely due to high expression levels of EGF within these exosomes, which activated the PI3K/AKT signaling pathway. These findings thereby demonstrate that PPY‐CHI/hEMSC‐Exo could serve as a potential treatment against MI to promote cardiac tissue repair, improve long‐term functioning, as well as alleviate arrhythmia.

## 5 Experimental Section

### Isolation, Culturing, and Examining the Differentiation Potential of hEMSCs

Human endometrial tissue was collected from 42 premenopausal women (mean age 47.21±5.1 [30–52] years), who underwent hysterectomies for uterine fibroids or adenomyosis, and were without administration of exogenous sex hormones for at least 3 months prior to surgery,^[^ ^12a^ ^]^ at the First Hospital of Shanxi Medical University, Taiyuan, China. All procedures were approved by the Medical Research Ethics Committee of Shanxi Medical University (REB #2 018 026 and SYDL2022006). Written informed consent was obtained from all patients.

Single cells were isolated from the endometrial tissue, as previously described.^[^ ^12^, ^33^ ^]^ Briefly, the endometrium was minced and digested with a mixture of enzymes, including collagenase type 3 (300 µg mL^−1^; Worthington Biochemical Corp, Freehold, NJ) and 40 µg mL^−1^ of deoxyribonuclease type I (Worthington Biochemical Corp.) for 45 min. The resulting dissociated cell suspension was neutralized within DMEM/Ham's Nutrient Mixture F‐12 (12 400 024, Gibco), supplemented with 100 U mL^−1^ penicillin, 100 µg mL^−1^ streptomycin, and 10% fetal bovine serum (FBS; 10099141C, Gibco). The single cells obtained were cultured and expanded via 3–6 passages for further studies. hEMSCs were identified using the Human MSC Analysis Kit (562 245, BD) with flow cytometry (FACSCelesta, BD, USA), and their differentiation capabilities were assessed by culturing them in differentiation media for either 12, 14, or 24 days to examine the occurrence of, respectively, osteoblastic, chondrogenic, or lipogenic differentiation, as previously documented.^[^ ^12a^ ^]^

### Isolation, Identification, and Measuring uptake of hEMSC‐Exo

To obtain hEMSC‐Exo, hEMSCs from passages 3–6 (P3‐6) were grown until cell confluence reached around 80%, and a satisfactory logarithmic growth stage was present. These cells were then washed 3 times with PBS, followed by culturing in serum‐free media for 48 h. Afterward, the conditioned medium was collected and centrifuged at 300 × g for 10 min, at 4 °C. The resulting supernatant was centrifuged again at 2000 × g for 20 min, at 4 °C, then at 10 000×g for 30 min to remove any residual cell debris, and filtered using a 0.22 µm membrane. This supernatant was then centrifuged (L‐100XP, Beckman USA) at 100 000 × g at 4 °C for 70 min to precipitate the hEMSC‐Exo, which were resuspended in 50–100 µL PBS and stored at −80 °C.

To characterize hEMSC‐Exo, total protein concentration was measured using a BCA protein concentration assay kit (SW101‐02, Seven Biotech), while the morphology was examined using TEM (JEOL‐100cx, JEOL Japan). NTA was used to determine the size distribution (ZataView, Particle Metrix, Germany). Western blot was used to identify hEMSC‐Exo surface markers, as described in “Western blot” below.

To measure hEMSC‐Exo uptake, exosomes (200 µg mL^−1^) were labeled with 5 µg mL^−1^ CM‐Dil (C7000, Invitrogen), and incubated with rat H9c2 cells (IMMOCELL, Xiamen Immocell Biotechnology Co., Ltd) for 6 h. Afterward, the supernatant was removed, and cells fixed with 4% paraformaldehyde. Nuclei were stained with DAPI (D9542, Sigma‐Aldrich), and hEMSC‐Exo uptake was observed using a fluorescent microscope (TI2‐U, Nikon, Japan).

### ROS and Hypoxic Treatment of H9c2 Cells

Rat H9c2 cells (IMMOCELL, Xiamen Immocell Biotechnology Co., Ltd) were cultured in DMEM/high glucose (12 800‐058, Gibco), containing 10% FBS, 100 U mL^−1^ penicillin, and 100 µg mL^−1^ streptomycin, to P3 for subsequent experiments. To stimulate ROS exposure, these cells were seeded in a 6‐well plate, at 8.0 × 10^5^ cells per well. Upon reaching 80–90% confluence, they were stimulated with 900 µm H_2_O_2_ for 4 h, as previously described.^[^ ^34^ ^]^ The resulting apoptosis rate was measured by flow cytometry. In brief, cells were digested with 0.1% trypsin (T4799, Sigma), centrifuged at 1000 × g for 5 min, suspended in 500 µL of 1X binding buffer with 5 µL annexin V‐FITC and 5 µL propidium iodide (MAO220‐1, Meilunbio), and incubated in the dark for 20 min.

To examine the effects of EGFR inhibitors, H9c2 cells, after exposure to H_2_O_2_, were incubated with 25 nm EGFR inhibitor for 24 h. As for the cotreatment group, those cells were incubated with EGFR inhibitor+10 µg mL^−1^ hEMSC‐Exo for 24 h.

With respect to hypoxia, H9c2 cells were subjected to hypoxia (0.1% O_2_) conditions, in the presence or absence of hEMSC‐Exo or EGFR inhibitor for 24 h. Additionally, H9c2 cells under hypoxic conditions were cultured on either PPY‐CHI or PPY‐CHI/hEMSC‐Exo‐coated dishes for 24 h.

### Hypoxic Treatment of HUVECs and Matrigel Tubule Assay

HUVECs were cultured under the same conditions as rat H9c2 cells. Matrigel tubule formation was then used to assess the proangiogenetic potential of hEMSC‐Exo in vitro. Matrigel (356 234, Corning) was thawed and placed into a 96‐well plate at 37 °C for 1 h to solidify. HUVECs were seeded into Matrigel‐coated wells at 3 × 10^4^ cells per well, and incubated with 400 µmol L^−1^ CoCl_2_ (P611644, Chengdu Huaxia Chemical Regent Co., Ltd) for 24 h to induce hypoxia; the control group was left untreated, Afterwards, the cells were incubated with hEMSC‐Exo (200 µg mL^−1^) for 3.5 h. The resulting tubule structures were photographed, and the number of tubes formed was counted by ImageJ.

### Measuring Cell Viability Using CCK‐8 Assay

Cell viability for H9c2 cells and HUVECs was measured using the CCK‐8 assay, following the manufacturer's instructions (Tocris Bioscience). Briefly, cells, after culturing for 24 or 48 h on uncoated control, CHI, PPY‐CHI, or PPY‐CHI/hEMSC‐Exo‐coated dishes, were incubated with 10 µL of CCK‐8 kit solution for 4 h, and the absorbance was measured at 450 nm using a microplate reader (Thermo Fisher).

### Synthesis and Characterization of PPY‐CHI Hydrogel

The conductive biomaterial PPY‐CHI was synthesized, as previously described.^[^ ^5^, ^6^, ^15^ ^]^ Briefly, a chemical oxidative polymerization method was used for PPY‐CHI (3:10 ratio) hydrogel synthesis, in which 60 µL of 98% pyrrole (Cat No.: 131 709, Sigma) was added to 10 mL of 2% CHI (Cat No.: 448 869, Sigma) acid solution. Oxidative polymerization was then carried out by adding FeCl_3_·6H_2_O (0.18 g, Cat No.: 236 489, Sigma) to the solution, followed by mixing for 48 h. Unpolymerized pyrrole and free Fe was removed using a dialysis membrane (Cat No.: 132 700, Fisher Scientific). CHI (2%) was used as a control. Both PPY‐CHI and CHI pH were adjusted to 6.0 using glycerol phosphate disodium (Cat No.: G9422, Sigma), and glutaraldehyde (Cat No.: G6257, Sigma) was used to form hydrogel.

FT‐IR (Bruker, INVENIO, Rheinstein, Germany) was used to assess the conjugation of PPY on CHI. PPY, PPY‐CHI, and CHI gels were lyophilized, ground into powder, and pressed onto glass sides to form sheets, at a pressure of 10 tons cm^−2^. These sheets were then scanned by FT‐IR, with a resolution of 4 cm^−1^, and 64 scans being taken. The range covered by these scans was 0–4000 cm^−1^, and each group was measured 3 times.

To examine the conductive properties of PPY‐CHI, PPY‐CHI, CHI control, and PPY‐CHI/hEMSC‐Exo hydrogels were placed on a platform to form films with a thickness of 1 mm. These films were positioned in close contact with 4‐point conductive analyzer probes; the probes were 2.5 mm apart from each other. The resistance of the hydrogels was measured using a 4‐wire resistance method (FT‐331, RuiKe, China), and 3 different areas of the membrane were tested.

### Incorporation of hEMSC‐Exo into PPY‐CHI and Characterization of Sustained Release

To incorporate hEMSC‐Exo into the PPY‐CHI hydrogel, 480 µL PPY‐CHI, 20 µL glycerol phosphate disodium (50% mass concentration), and 40 µL hEMSC‐Exo (2 mg mL^−1^), and 6.5 µL glutaraldehyde (0.04%) were successively added, and vortexed. The gelation time could be altered by varying the glutaraldehyde concentration, though 0.04% glutaraldehyde is similar to that used for preparing bioprosthetic valves employed in clinical practices.^[^ ^35^ ^]^

To characterize the sustained release of the resulting PPY‐CHI/hEMSC‐Exo hydrogel, protein release and quantification experiments were carried out, in which PPY‐CHI/hEMSC‐Exo, at an exosome concentration of 200 µg mL^−1^, was placed into a 35 mm Petri dish. A dish with the same amount of PBS served as the control, and both dishes were incubated at 37 °C for 15 min. Subsequently, 500 µL PBS was added to both dishes, and changed daily. The resulting supernatant was then collected and centrifuged at 7000 rpm for 5 min, and total amount of hEMSC‐Exo protein was measured using the BCA method. Additionally, the release of the encapsuled hEMSC‐Exo from the hydrogel was visualized using TEM.

### Establishing the Rat MI Model and Functional Assessments

Female Sprague–Dawley rats (80; each 220–250 g) were purchased from the Animal Center of Shanxi Medical University (SCXK, 2019‐0004). All animal procedures were approved by the Animal Care Committee of Shanxi Medical University, and were carried out in accordance with the Guide for the Care and Use of Laboratory Animals (NIH, 8th Edition, 2011).

All rats were anesthetized with 2% isoflurane (R510‐22, RWD), and MI was induced by exposing the anterior wall of the left ventricle, via a 4–5 anterior intercostal oblique incision on the left chest, followed by ligation of the left anterior descending coronary artery. ECG was used to identify the characteristic ST‐segment elevation. Rats were then randomly divided into 4 groups: PBS control, hEMSC‐Exo, PPY‐CHI, and PPY‐CHI/hEMSC‐Exo. Four weeks later, intercostal thoracotomy was then carried out among these rats and 100 µL of PBS, hEMSC‐Exo (80 µg per rat, comparable to the release from 2 × 10^6^ hEMSCs),^[^ ^36^ ^]^ PPY‐CHI or PPY‐CHI/hEMSC‐Exo was injected into the infarct site.

Cardiac function was assessed using echocardiography, prior to MI, 4 weeks post‐MI, as well as 1 and 4 weeks postinjection. FS, LVIDs, and LVIDd were measured (GE Vivid7, US).

Additionally, at 4 weeks postinjection, cardiac electrophysiological properties, particularly in terms of responses to induced arrhythmias, were then assessed using PES of the left ventricle, based on methods outlined by He et al.^[^ ^15^ ^]^ In brief, rats were anesthetized with 2% sevoflurane (22 011 031, Hearem), followed by subcutaneous placement of the electrodes and recording of surface ECGs with an electrophysiological recorder (PE231 and PL3508LL2, AD Instruments, Australia). Thoracotomy was then performed to expose the epicardium, and 2 stimulating electrodes were inserted, one at the right ventricular outflow tract, and another at the left ventricular apex. Ventricular arrhythmia was induced using a programmed electrophysiological stimulator (KST 2‐CH, Kexin Medical Biotechnology Co., Ltd., Shanghai, China), which first involved a basic cycle of 120 ms (S0), followed by single (S1,70 ms), double (S2, 60 ms), and triple (S3, 50 ms) stimulation at shorter coupling intervals. This cycle was repeated over the course of 10 min, and the corresponding ECG waveform changes were recorded (Lab Chart8.0, AD Instruments, Australia). The severity of the resulting arrhythmias was scored based on a previously‐described inducing quotient,^[^ ^15^ ^]^ ranging from 0 to 7; the higher the score, the more severe the arrhythmia.

### Histological, Immunofluorescent, and TUNEL Staining

To obtain cardiac tissue sections for histological and immunofluorescent staining, rats were sacrificed at 28 days postinjection; their hearts were fixed for 48 h in 4% paraformaldehyde, and cut along the transverse plane to obtain rings 2 mm thick. These rings were then dehydrated, paraffin‐embedded, and sectioned into 3 µm sections. The extent of fibrosis present in these hearts was determined by staining these sections with Masson's trichrome stain (PH1427, Phygene), followed by imaging under a microscope and quantification with ImageJ.

As for immunofluorescent staining, the tissue sections were de‐paraffinated and incubated with primary antibodies against α‐SMA (a5528, Sigma), first at 37 °C for 1 h, room temperature for 2 h, and finally overnight at 4 °C. Afterward, the slides were incubated with corresponding Alexa 488‐conjugated secondary antibodies (A1108, Invitrogen; 1:1000) for 1 h at room temperature in the dark. Nuclei were stained with DAPI, and a Nikon fluorescence inverted microscope was used to observe the sections.

To determine the extent of apoptosis in the heart, TUNEL staining was conducted, following the manufacturer's instructions (MK1019, BOSTER). Nuclei were stained with DAPI, and apoptotic cells quantified using ImageJ.

### Western Blot

To determine protein expression levels, total protein was extracted from cells or heart tissue (at MI site and surrounding regions), and standard procedures were followed, as previously described.^[^ ^37^ ^]^ The antibodies used were as follows: AKT, p‐AKT, PI3K, p‐PI3K (respectively, 4691S, 4060S, 4257S, 4228S, Cell Signaling Technology), BAX (SC‐20067, Santa Cruz Biotechnology), and BCL‐2 (68103‐1‐Ig, Proteintech). All of the aforementioned protein expression levels were normalized to GAPDH (MAB374, Millipore). Additionally, antibodies against exosome markers TSG101 (28283‐1‐AP, Proteintech), CD63 (MA5‐30187, Invitrogen), and CD81 (27855‐1‐AP, Proteintech) were used.

### Identification of hEMSC‐Exo Proteins with Human Cytokine Antibody Array

After total hEMSC‐Exo protein was measured using BCA assay, 1 mL of the supernatant was diluted in a blocking buffer, to yield a protein concentration of 250–300 µg mL^−1^, which was then placed on the chip (ab133998, Abcam), and incubated, first at room temperature for 2 h, followed by overnight at 4 °C. The chip was washed 3 times at room temperature with 1X Wash Buffer I, then II, each for a duration of 10 min, followed by incubation with 1 mL of biotin‐conjugated anticytokine antibodies at room temperature for 2 h. After washing 3 times for 10 min, the chip was incubated with HRP‐conjugated streptavidin at room temperature for 2 h. Equal volumes of Detection Buffers C and D were mixed together, and 500 µL of the mixture was added dropwise to each chip, followed by incubation at room temperature for 2 min. The amount of resulting chemiluminescence was quantified by ImageJ.

### Sequencing of hEMSC‐Exo RNAs

Total RNA from hEMSC‐Exo was extracted using Trizol reagent (10 296 028, Invitrogen), and purified using the Total RNA Purification Kit (TRK‐1001, LC Sciences), in line with the manufacturer's instructions. RNA was then quantified using NanoDrop (ND‐2000, Thermo Scientific, USA), and integrity tested by Bioanalyzer (2100, Agilent Technologies, USA), at concentrations of >50 ng µL^−1^ (RIN > 6.3, OD260/280>1.8, total RNA>1 µg). A miRNA library was constructed using the TruSeq Small RNA Sample Prep Kit (RS‐200‐0012, Illumina), which entails ligation of 3′ and 5′ linker sequences, reverse transcription of RNAs to yield cDNA, PCR amplification of the cDNA, isolation of the target fragments via electrophoresis, and machine sequencing, with a read length of 1 × 50 bp (Hiseq2500, Illumina, USA).

### Statistical Analysis

Data were expressed as mean ± standard deviation, and found to exhibit a normal/Gaussian distribution under the Shapiro–Wilk test. Analyses were performed using GraphPad Prism software (v. 9.3.1). Unpaired Student's *t*‐test (2‐tailed) was performed for comparisons between 2 groups, while for 3 or more groups, either one‐way analysis of variance (ANOVA), or two‐way ANOVA with repeated measures over time, followed by, respectively, Tukey's or Bonferroni post hoc tests. The sample size for each analysis was presented within the figure legends. *p* < 0.05 were considered statistically significant.

## Conflict of Interest

The authors declare no conflict of interest.

## Supporting information

Supporting Information

## Data Availability

The data that support the findings of this study are available from the corresponding author upon reasonable request.
