# Supplementary material for: A Novel Conductive Polypyrrole‐Chitosan Hydrogel Containing Human Endometrial Mesenchymal Stem Cell‐Derived Exosomes Facilitated Sustained Release for Cardiac Repair
Source: Adv Healthc Mater. 2024 Jan 14;13(10):2304207. doi: 10.1002/adhm.202304207 (PMC11468178; doi:10.1002/adhm.202304207)
Supplement: Supplementary file 1 — Supporting Information [file ADHM-13-2304207-s001.pdf]

ADVANCED HEALTHCARE MATERIALS

Supporting Information

for *Adv. Healthcare Mater.*, DOI 10.1002/adhm.202304207

A Novel Conductive Polypyrrole-Chitosan Hydrogel Containing Human Endometrial Mesenchymal Stem Cell-Derived Exosomes Facilitated Sustained Release for Cardiac Repair

Changping Yan, Xinzhu Wang, Qi Wang, Haiyan Li, Huifang Song, Jingli Zhou, Zexu Peng, Wenjuan Yin, Xuemei Fan, Kun Yang, Bingrui Zhou, Yuxiang Liang, Zengyu Jiang, Yuwei Shi, Sanyuan Zhang, Sheng He*, Ren-Ke Li* and Jun Xie**

Supplementary Material

for

A Novel Conductive Polypyrrole-Chitosan Hydrogel Containing Human Endometrial Mesenchymal Stem Cell-Derived Exosomes Facilitated Sustained Release for Cardiac Repair

Changping Yan[#], Xinzhu Wang[#], Qi Wang[#], Haiyan Li, Huifang Song, Jingli Zhou, Zexu Peng,

Wenjuan Yin, Xuemei Fan, Kun Yang, Bingrui Zhou, Yuxiang Liang, Zengyu Jiang, Yuwei Shi,

Sanyuan Zhang, Sheng He[*], Ren-Ke Li[*], and Jun Xie[*]*

C. Yan, X. Wang, Q. Wang, H. Li, H. Song, Z. Peng, W. Yin, X. Fan, K. Yang, B. Zhou, Y. Liang, Z. Jiang, Y. Shi, S. Zhang, S. He, J. Xie

The First Hospital of Shanxi Medical University, Department of Biochemistry and Molecular Biology, Shanxi Key Laboratory of Birth Defect and Cell Regeneration, MOE Key Laboratory of Coal Environmental Pathogenicity and Prevention, Shanxi Medical University, Taiyuan, China, 030001

Email: zsyprofessor@aliyuan.com; hesheng@sxmu.edu.cn; junxie@sxmu.edu.cn

C. Yan

Department of Gynecology, Affiliated Cancer Hospital of Shanxi Medical University, Taiyuan, China

H. Song

Department of Anatomy, Shanxi Medical University, Taiyuan, China

J. Zhou

Shanxi Provincial People's Hospital, Affiliated Hospital of Shanxi Medical University, Taiyuan, China

Y. Shi

NHC Key Laboratory of Pneumoconiosis; Shanxi Province Key Laboratory of Respiratory; Department of Pulmonary and Critical Care Medicine, The First Hospital of Shanxi Medical University

R-K. Li

Toronto General Hospital Research Institute, Division of Cardiovascular Surgery, University Health Network, University of Toronto, Toronto, ON, Canada, M5G 2C4

Email: Ren-Ke.Li@uhnresearch.ca

[#]These authors contributed equally

Supplementary Figures

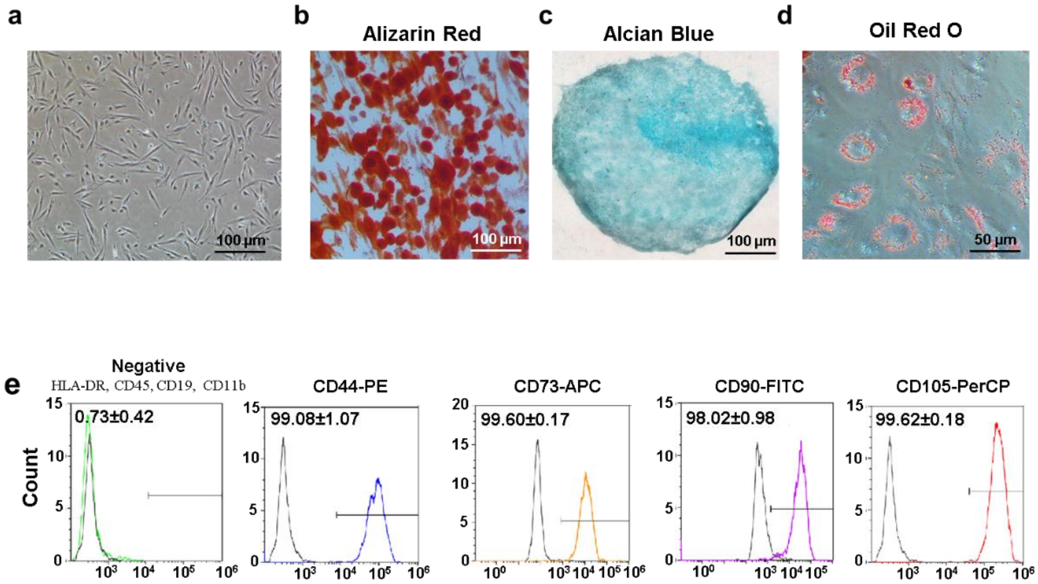

Figure S1. Identification and differentiation of human endometrial mesenchymal stem cells (hEMSCs). (a) Representative micrograph of hEMSCs from premenopausal donors (30-50 years). (b) Representative micrograph of osteogenic differentiation, indicated by Alizarin red staining of differentiated osteocytes. (c) Representative micrograph of chondrogenic differentiation, indicated by Alcian blue staining of differentiated chondrocytes. (d) Representative micrograph of adipogenic differentiation, indicated by oil Red O staining of lipid droplets. (e) Flow cytometry results indicating the presence of mesenchymal stem cell surface markers CD44, CD73, CD90, and CD105 on hEMSCs.

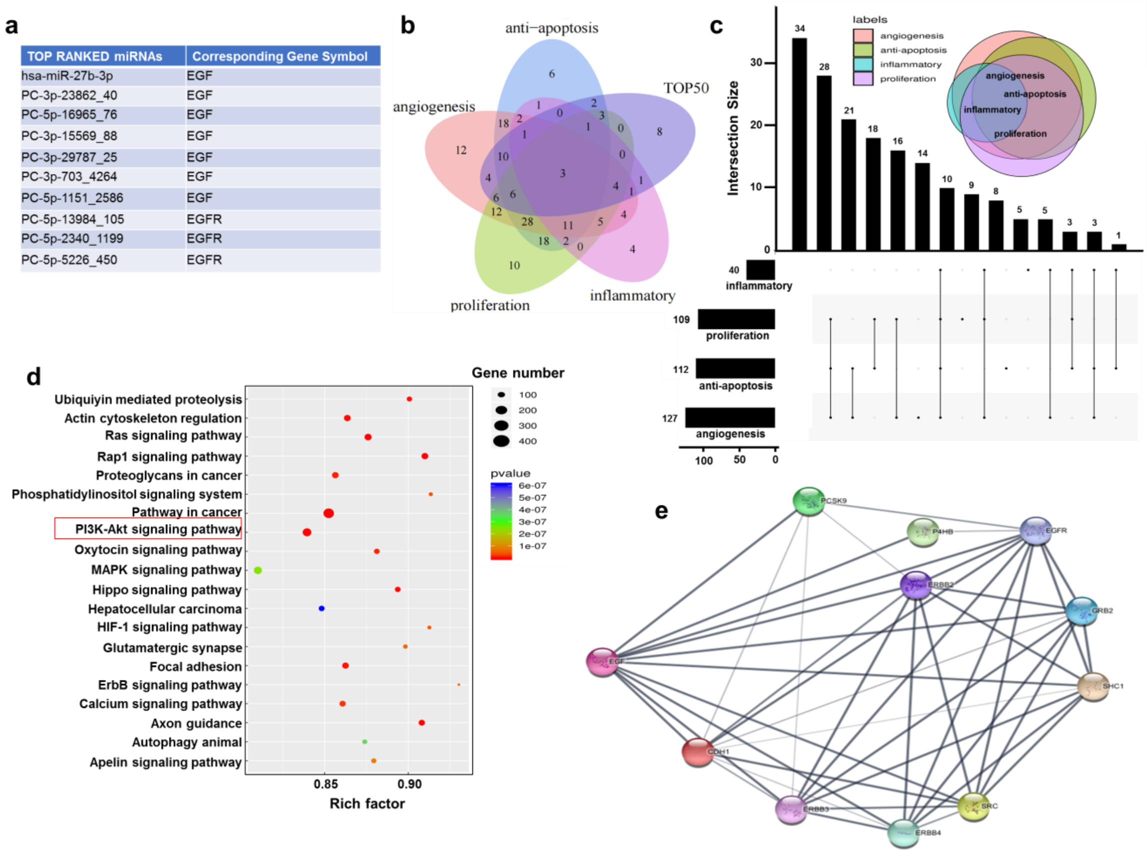

Figure S2. Identification of the top 50 most expressed miRNAs in hEMSC-derived exosomes (hEMSC-Exo) and their associated signaling pathways. (a) Table showing the top 10 most-expressed miRNAs out of the top 50, which were associated with epidermal growth factor (EGF) and receptor (EGFR) proteins. (b) Euler diagram showing the functional classification of the top 50 miRNAs into anti-apoptosis, angiogenesis, proliferation, and inflammatory categories. (c) Representative inflammatory, proliferation, anti-apoptosis and angiogenesis miRNAs, and their intersection sizes, based on transcriptome sequencing results. (d) Kyoto Encyclopedia of Genes and Genomes analysis showing the most enriched pathways for hEMSC-Exo miRNAs. (e) EGF Protein-protein interaction network generated from STRING.

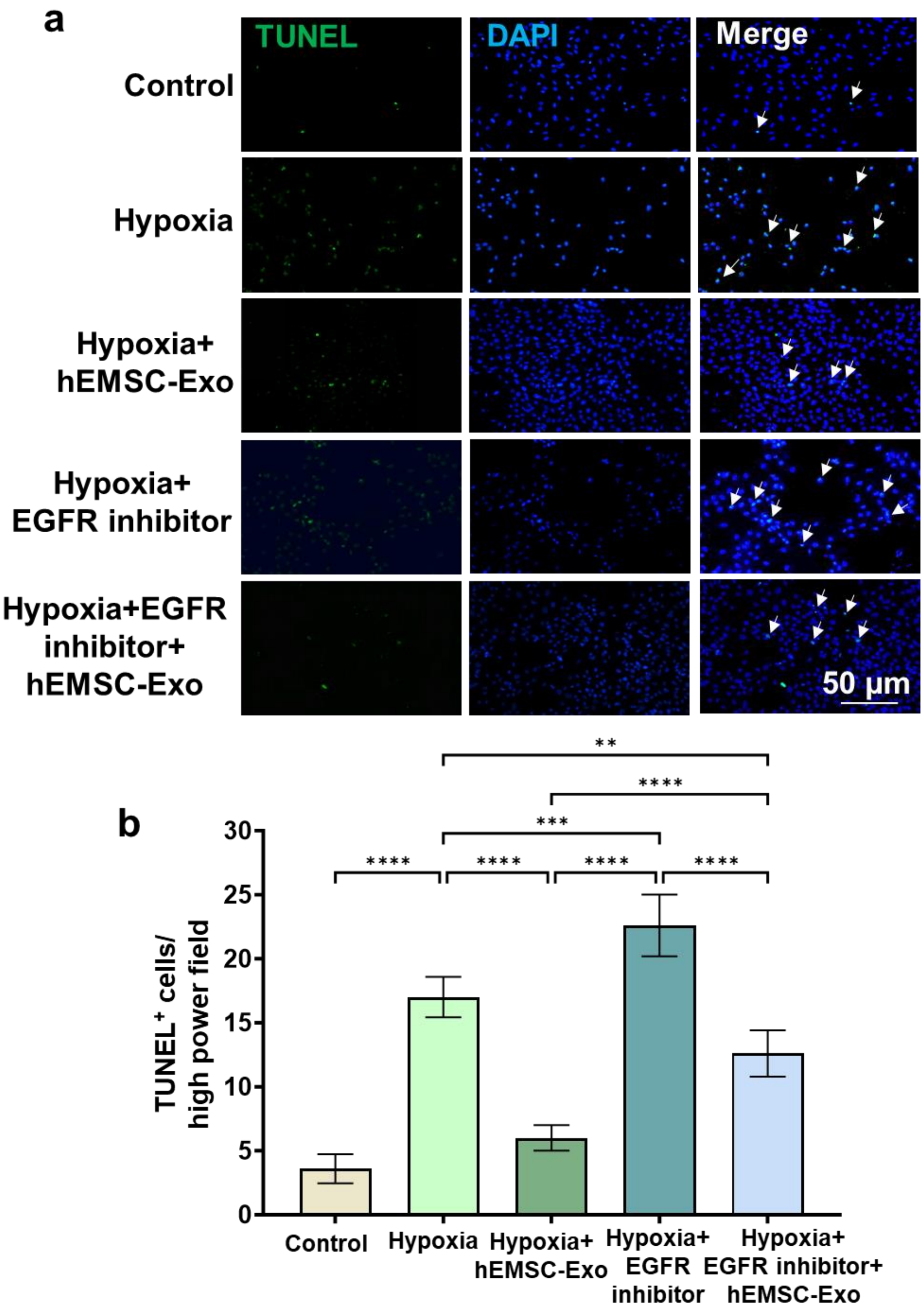

Figure S3. hEMSC-Exo exerts cyto-protective effects under hypoxic conditions (a) Representive immunofluorescence images and (b) quantification for $TUNEL^+$ H9c2 cells among untreated control (Control), hypoxic (Hypoxia), Hypoxia+hEMSC-Exo, Hypoxia+EGFR inhibitor, and Hypoxia+EGFR inhibitor+hEMSC-Exo groups. Data are expressed as mean ± SD. n=5/group for all experiments. **p<0.01, ***p<0.001, ****p<0.0001.

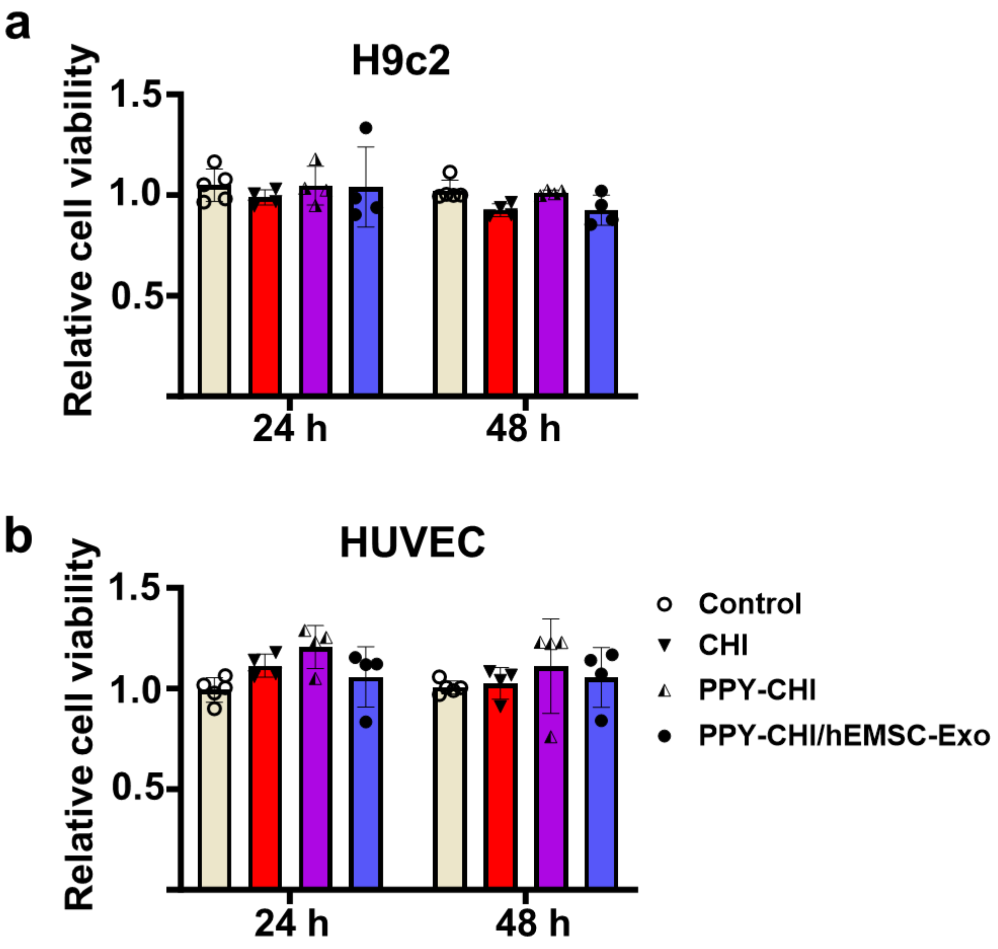

Figure S4. H9c2 and human umbilical cord vein endothelial (HUVEC) cell viability on chitosan (CHI), polypyrrole-conjugated chitosan (PPY-CHI), and PPY-CHI with hEMSC-Exo (PPY-CHI/hEMSC-Exo)-coated dishes. Relative cell viabilities among control, CHI, PPY-CHI, and PPY-CHI/hEMSC-Exo-coated dishes for (a) H9c2 cells and (b) HUVECs, after 24 and 48 hours of culturing. n=4-5/group for all experiments.

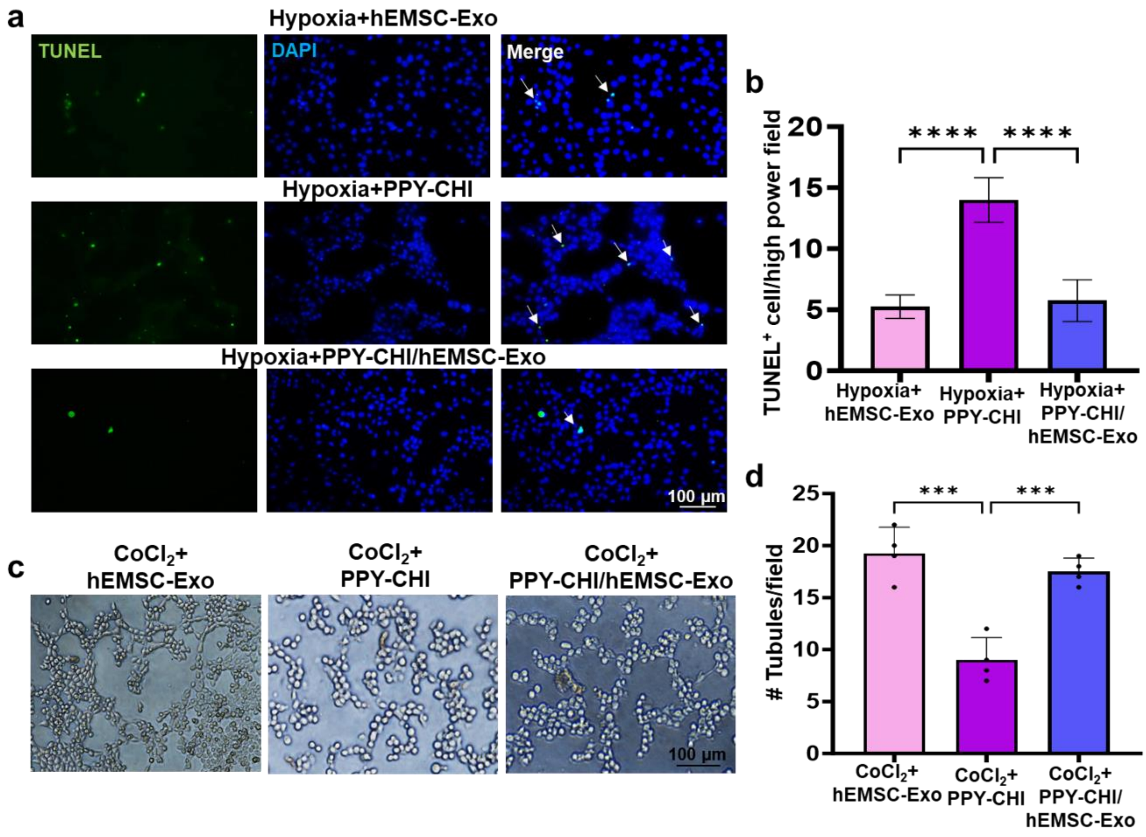

Figure S5. hEMSC-Exo exerts cyto-protective effects *in vitro* on H9c2 cells and promoted HUVEC tube formation. (a) Representative immunofluorescence images and (b) quantification for TUNEL$^+$ H9c2 cells among Hypoxia+hEMSC-Exo, Hypoxia+PPY-CHI, and Hypoxia+PPY-CHI/hEMSC-Exo groups. (c) Representative micrographs and (d) quantification of Matrigel tube formation by HUVECs among $CoCl_2$ (hypoxia inducer, 400 μmol/L)+hEMSC-Exo, $CoCl_2$+PPY-CHI, and $CoCl_2$+PPY-CHI/hEMSC-Exo groups. Data are expressed as mean ± SD. n=4/group for all experiments. ***$p<0.001$, ****$p<0.0001$.
